# A Study of Super Refractory Status Epilepticus from India

**DOI:** 10.3389/fneur.2017.00636

**Published:** 2017-11-28

**Authors:** Usha K. Misra, Jayantee Kalita, Deepanshu Dubey

**Affiliations:** ^1^Department of Neurology, Sanjay Gandhi Postgraduate Institute of Medical Sciences, Lucknow, India

**Keywords:** super refractory status epilepticus, status epilepticus, refractory status epilepticus, non-convulsive status epilepticus, metabolic encephalopathy

## Abstract

**Background:**

Super refractory status epilepticus (SRSE) is an important and recently recognized neurological emergency.

**Purpose:**

In view of paucity of studies on SRSE, we report the frequency, etiology and outcome of SRSE.

**Methods:**

In a hospital-based observational study during 2013 to 2016, consecutive patients with SRSE [persistence of status epilepticus (SE) for 24 h or more, or recurrence of SE on weaning of intravenous anesthetic] were included. The demographic, clinical, and laboratory data were obtained and the severity of SE was defined using Status Epilepticus Severity Score (STESS). The outcome was defined as control of SE, hospital death, and functional status at the time of discharge.

**Results:**

Fourteen (13%) patients developed SRSE. Their median age was 27.5 (2–70) years and four were below 18 years of age. The etiology of SRSE was metabolic encephalopathy and encephalitis in five patients each, cerebral venous sinus thrombosis in one and miscellaneous disorders in three patients. Six (43%) patients died. The patients with SRSE had higher admission STESS (*p* = 0.04), and longer intensive care unit (*p* < 0.01) and hospital (*p* = 0.004) stay compared to non-SRSE group. The patients with treatable etiology had better outcome.

**Conclusion:**

SRSE occurred in 13% patients with SE and 43% of them died. The SRSE patients with treatable etiology had a better outcome.

## Highlights

- SRSE occurs in 13% of patients with status epilepticus.
- SRSE has a mortality of 43%.
- The SRSE patients with a treatable etiology have a better outcome.

## Introduction

Super refractory status epilepticus (SRSE) is defined as continuous or recurrent seizures without normalization of consciousness lasting for 24 h or more despite administration of an intravenous (IV) anesthetic (midazolam, propofol, ketamine, or barbiturate), or recurrence of status epilepticus (SE) on weaning of IV anesthetics (1). In a prospective study, 9% (12/128) of SE episodes failed second-line antiepileptic drugs (AEDs) and required coma induction. Persistence or recurrence of seizures after 24 h following treatment were not mentioned in this study; hence some of these patients may have not fulfilled the proposed criteria of SRSE (2). The term SRSE was introduced in 2011 in London-Innsbruck Colloquium; therefore, there is paucity of information on SRSE (1). SRSE is more likely following acute brain injury rather than chronic epilepsy and is more likely after central nervous system (CNS) infection, stroke, or in a healthy brain with new onset refractory status epilepticus (3). The predictors of refractory status epilepticus (RSE) are acute SE etiology, coma/stupor, and serum albumin <3.5 g/l (4). Development of RSE or SRSE was associated with stupor or coma on admission and absence of history of epilepsy. These patients had a worse outcome compared to those who responded to AEDs (5). Infections can also complicate SE and are associated with a poor outcome (6). The etiology of SRSE in the developing countries is dominated by CNS infections (7). SRSE can be convulsive or non-convulsive. The convulsive SE can result in serious complications such as myocardial injury, aspiration pneumonia, pulmonary edema, and rhabdomyolysis leading to renal failure. The diagnosis of non-convulsive SE (NCSE) requires electroencephalography (EEG), which may delay the diagnosis. SE may occur due to failure of the mechanisms responsible for seizure termination or from the initiation of mechanisms leading to prolonged seizures (t1). Persistence of SE can cause long-term sequelae (after time point t2) including neuronal injury, neuronal death, and alteration of neuronal network depending on the type and duration of seizures. For convulsive SE, t1 is 5 min and t2 is 30 min. For other types of seizures, t1 and t2 are not known (8). There is paucity of studies on SRSE, and the available information is based on retrospective review of cases. From China, 13 patients with SRSE were reported during January 2010 to August 2013 (9); from France, 78 patients were collected in an intensive care unit (ICU) survey during 2001 to 2011 having SE more than 7 days of anesthesia (10), and from South India (Hyderabad) 30 (16.9%) patients, and from Vellore 17 (7.7%) events of SRSE were reported (11, 12). In a recent population-based survey from Germany, during 2008 to 2013, 338 out of 2,585 (13%) patients with SE had SRSE (13). In the present study, we report the frequency, etiology, predictors, and prognosis of SRSE.

## Subjects and Methods

This retrospective analysis of patients with SRSE was conducted at a tertiary care teaching hospital in India during September 2013 to January 2016. The patient information was retrieved from a prospectively maintained SE registry. The project was approved by the Ethics Committee of Sanjay Gandhi Post Graduate Institute of Medical Sciences (N0 2017/161/IP/EXP).

### Definitions

Status epilepticus was defined as continuous convulsion lasting 5 min or more or recurrent seizures without regaining consciousness to baseline between the attacks. Subtle SE was defined as the presence of continuous ictal discharges on EEG along with subtle convulsive movements (14). RSE was defined if SE persisted despite adequate dose of benzodiazepines and another AED (14). SRSE was defined as continuous or recurrent seizures without normalization of consciousness for 24 h or more despite administration of IV anesthetic agent (midazolam, propofol, ketamine, or phenobarbital) or recurrence of SE on weaning of IV anesthetic (1).

### Encephalitis

The patients with fever, altered consciousness and cerebrospinal fluid (CSF) pleocytosis were diagnosed as encephalitis in whom septic or fungal meningitis and malaria were excluded.

### Encephalopathy

The patients with altered consciousness, normal CSF and abnormal biochemical tests were diagnosed as metabolic encephalopathy.

### Evaluation

Demographic information, history of epilepsy, drug default, and precipitating causes were noted. Consciousness was assessed by Glasgow Coma Scale. SE was categorized into generalized convulsive SE or NCSE. NCSE was diagnosed when there was change in consciousness or behavior from the baseline for more than 30 min with EEG showing epileptiform discharges or rhythmic theta or delta activity (>2 Hz), temporal evolution of epileptiform, or rhythmic activity at more than 1 Hz with change in location and frequency or improvement in clinical and EEG findings following intravenous AEDs (15). The severity of SE was determined by the Status Epilepticus Severity Score (STESS). A STESS score of 0–2 was considered favorable and 3–6 as unfavorable (16). The etiology of SE was classified into acute symptomatic, remote symptomatic, progressive symptomatic, idiopathic, or cryptogenic (17). The etiology of SRSE was further classified as potentially fatal (large infarction, intra-cerebral hemorrhage, CNS or systemic infection, acquired immunodeficiency syndrome, metabolic abnormalities like chronic renal failure on dialysis, poisoning, vasculitis, brain tumor, or neurosurgery) or acute symptomatic etiology with a favorable outcome (drug withdrawal) (18). Pre-hospital treatment was noted and the duration of SE and comorbidities were recorded. Duration of ICU stay, mechanical ventilation (MV) and ICU complications were also noted.

### Investigations

Blood counts, blood urea nitrogen, serum chemistry (glucose, creatinine, transaminases, bilirubin, and electrolytes), and arterial blood gas (ABG) abnormalities were noted. EEG was done within 12 h of admission. In convulsive SE, the EEG was recorded after clinical cessation of SE if the consciousness did not return to the baseline. Cranial computerized tomography or magnetic resonance imaging (MRI) was done as soon as possible after stabilizing the patient. CSF examination was done if CNS infection was suspected. The etiology of SE was also categorized into metabolic encephalopathy, stroke, CNS infections, and miscellaneous groups.

### Management

All the patients with SE were treated as per a predefined protocol with lorazepam 0.1 mg/kg (maximum 4 mg) IV, which was repeated if the seizures were not controlled within 10 min. Controlled and time linked escalation of AEDs was done in the resistant patients. The patients were intubated and mechanically ventilated if ABG analysis revealed hypoxia (PaO_2_ <60 cm of H_2_O), hypercarbia (PaCO_2_ >52 cm H_2_O) or acidosis (pH <7.3) (19). Burst suppression was done in few. The anesthetic drugs were tapered after 48 h of seizure freedom and were reintroduced if there was a recurrence of SE.

Outcome was defined as death or disability at the time of discharge using modified Rankin’s scale (mRS).

### Statistical Analysis

The demographic, clinical, laboratory findings, and outcome of SRSE were compared with RSE and SE groups using parametric tests for categorical and non-parametric tests for continuous variables. The outcome in different etiological groups was compared using chi square test. The statistical tests were done employing SPSS 18 version software. A variable having two-tailed *p*-value of ≤0.05 was considered significant.

## Results

Fourteen out of 107 (13.1%) patients with SE developed SRSE. The median age of the patients with SRSE was 27.5 (2–76) years; four below 18 years of age and seven were females. Metabolic etiology was present in five; acute renal failure in one, and chronic dialysis dependent in four patients. Comorbidities included hypertension in five patients, diabetes and chronic liver disease in one each. In five patients, SRSE was due to CNS infection; herpes simplex encephalitis (HSE) in two, scrub typhus in one, and nonspecific encephalitis in two. One patient had cerebral venous sinus thrombosis due to systemic lupus erythematosus. In the miscellaneous group, there were three patients; one with post-anoxic SE, one with posttraumatic arteriovenous fistula which was treated with local onyx glue injection, and another had growth retardation with hyper IgE syndrome. In the four etiologic groups of SRSE, the demographic features and duration of seizure before starting treatment were not significantly different. More than one comorbidity was present in four (28.6%) patients. The MRI findings in the encephalitis group revealed characteristic frontotemporal involvement in the patients with HSE. In the metabolic group, MRI was abnormal in two patients. In three patients, the MRI changes were attributed to SE *per se*.

### Management

Midazolam was administered to all the patients, propofol to five, and phenobarbital and ketamine to two patients each. Burst suppression was done in one patient using phenobarbital for 2 days. Ketogenic diet was prescribed to one patient, and SE was controlled after 3 days. The details of SRSE patients are presented in Table 1.

**Table 1:** Characteristics of the patients with super refractory status epilepticus.

Patient number	Age years	Etiology	SE duration (total in h)	SE duration before treatment (in h)	Cranial magnetic resonance imaging	Drugs given	Outcome (death/discharge)
1	60	scrub typhus	48	20	Normal	V, M, L	Discharge
2	20	Herpes simplex encephalitis (HSE)	240	3	BL temporal T2 Hyper intensities with right temporal arachnoid cyst	V, Lev, L, M, PHT, Ph, CBZ, Pr	Discharge
3	19	HSE	480	24	Bilateral frontotemporal T2 hyperintensities	V, Lev, L, M, T, CBZ, Pr	Discharge
4	32	Acute on CRF and mesenteric ischemia	26	1	Normal	V, Lev, L, M	Death
5	38	Encephalitis with thrombocytopenia with ARF	26	0.5	Normal	V, Lev, L, M, K	Death
6	40	Posttraumatic dural arteriovenous fistula	168	1	Arteriovenous fistula	V, Lev, L, CBZ, T, M, Pr	Discharge
7	13	Post-hypoxic	26	1	–	V, Lev, M	Death
8	13	Thrombotic thrombocytopenic purpura with ARF	26	0.5	BL asymmetric parieto occipital T2 hyperintensity	V, Lev, PHT, M, Pr, K	discharge
9	9	Encephalitis? Cause	504	4	BL laminar cortical necrosis and T2 Hyperintensity	V, Lev, PHT, M, CBZ, Ph, Z,	Discharge
10	76	CRF with encephalopathy	48	6	Multiple periventricular ischemic changes	V, Lev, M, L	Death
11	27	CRF with encephalopathy	120	1	BL laminar cortical necrosis	Lev, V, PHT, M, L	Discharge
12	28	CRF with encephalopathy	120	0.17	Normal	PHT, V, Lev, M	Death
13	33	Cerebral venous thrombosis with systemic lupus erythematosus	432	0.17	Hemorrhagic infarct bilateral frontal and right tempoparietal involvement	Lev, V, L, CBZ, M, T, Pr	Death
14	2	Global developmental delay with familial hyper IgE syndrome	384	1	Cortical laminar necrosis	Lev, PHT, M, CBZ	Discharge

*ARF, acute renal failure; BL, bilateral; CBZ, carbamazepine; CRF, chronic renal failure; K, ketamine; L, lacosamide; Lev, levetiracetam; M, midazolam; PHT, phenytoin; Ph, Phenobarbital; Pr, propofol; SE, status epilepticus; T, topiramate; V, valproate; Z, zonisamide*.

The duration of hospitalization did not significantly differ in various etiological subgroups of SRSE, except the encephalitis group had longer hospital stay compared to miscellaneous and metabolic groups. The mortality in the four etiological groups was also not significantly different, but encephalitis group had fewer deaths because of treatable etiology (HSE in two patients; Table 2).

**Table 2:** Characteristics of patients with super refractory status epilepticus in different subgroups.

	Metabolic (*n* = 5)	Encephalitis (*n* = 5)	Stroke (*n* = 1)	Miscellaneous (*n* = 3)
Age in years (median. range)	28 (13–76)	20 (9–60)	33	13 (2–40)
Gender (female)	2	3	1	1
Duration of SE hours (median, range)	48 (26–120)	240 (24–504)	432	168 (26–384)
Duration of SE before treatment hours (median, range)	1 (0.17–60)	4 (0.5–24)	0.17	1 (1)
STESS (favorable 0–2)	0	0	0	1
Comorbidities	5	1	1	0
Brain imaging (abnormal)	2	2	1	2
Death	3	1	1	1
Good outcome (mRS 0–3)	1	3	0	0
Duration of hospitalization days (median range)	22 (1–60)	27 (1–79)	45	25 (4–56)
Duration of MV days (median, range)	6 (1–60)	10 (1–38)	30	14 (4–31)
Duration of ICU stay in days (median, range)	8 (1–60)	14 (1–47)	40	21 (4–50)

*ICU, intensive care unit; mRS, modified Rankin Scale; MV, mechanical ventilation; SE, status epilepticus; STESS, status epilepticus severity score; VAP, ventilator associated pneumonia*.

### Predictors and Outcome of SRSE

Admission STESS (*p* = 0.04), duration of ICU stay (*p* < 0.01), and duration of hospitalization (*p* = 0.004) were significantly longer in the patients of SRSE compared to non-SRSE (Table 3). There was no difference between the groups of SE and SRSE with respect to the duration of SE before treatment (*p* = 0.70), age (*p* = 0.09), gender (*p* = 0.19), death (*p* = 0.16), and outcome at the time of discharge (*p* = 0.12). The details are presented in Table 3. The patients with SRSE had longer duration of SE (*p* < 0.01), ICU stay (*p* = 0.002), and MV (*p* < 0.01) compared to RSE (Table 4).

**Table 3:** Comparison of patients with status epilepticus (SE) and super refractory status epilepticus (SRSE).

	SE (*n* = 107)	SRSE (*n* = 14)	*p*–Value
Age (median, range) years	35 (2–90)	27.5 (2–76)	0.09
Female gender	37 (34.6%)	7 (50%)	0.19

**Etiology**			
Metabolic	23 (21.5%)	5 (35.7%)	0.17
CNS infections	30 (28%)	5 (35.7%)	0.49
Stroke	25 (23.4%)	1 (7.1%)	0.12
Drug withdrawal	4 (3.7%)	0 (0%)	0.42
Tumors	6 (5.6%)	0 (0%)	0.33
Others	19 (17.8%)	3 (21.4%)	0.06
Duration of SE hours (median, range)	4 (0.08–504)	120 (24–504)	<0.01
Duration before treatment in hours (median, range)	1 (0.08–160)	1 (0.17–24)	0.60
STESS (favorable 0–2)	32 (29.9%)	1 (7.1%)	0.046

**Outcome**			
Death	29 (27.1%)	6 (42.9%)	0.16
Good outcome (mRS 0–3)	59 (55.1%)	5 (35.7%)	0.12
Duration of hospitalization in days (median, range)	14 (1–79)	26 (1–79)	0.004
Duration of MV (median range) in days	0 (0–60)	12 (1–60)	<0.01
ICU stay (median, range) in days	2 (0–60)	17.5 (1–60)	<0.01
Hypotension	48 (45.8%)	13 (92.9%)	<0.01
VAP	24 (22.4%)	8 (57.1%)	<0.01
Sepsis	62 (57.9%)	11 (78.6%)	0.09
Arrhythmia	4 (3.7%)	1 (7.1%)	0.47
Tracheostomy	14 (13.1%)	6 (42.9%)	<0.01

*CNS, central nervous system; ICU, intensive care unit; MV, mechanical ventilation; STESS, status epilepticus severity score; VAP, ventilator associated pneumonia; mRS, modified Rankin’s scale*.

**Table 4:** Comparison of patients with refractory status epilepticus (RSE) and super refractory status epilepticus (SRSE).

	SRSE (*n* = 14)	RSE (*n* = 34)	*p*-Value
Age years (median range)	27.5 (2–76)	32.5 (3–90)	0.22
Female gender	7 (50.0%)	13 (38.2%)	0.45

**Etiology**			
Metabolic	5 (35.7%)	12 (35.3%)	0.98
CNS infections	5 (35.7%)	10 (29.4%)	0.67
Stroke	1 (7.1%)	7 (20.6%)	0.26
Drug withdrawal	0	1 (2.9%)	0.51
Tumors	0	0	
Others	3 (21.4%)	4 (11.8%)	0.10
Duration of SE hours (median, range)	120 (24–504)	12 (1–168)	<0.01
Duration before treatment hours (median. range)	1 (0.17–24)	1 (0.08–160)	0.29
STESS (favorable 0–2)	1 (7.1%)	8 (23.5%)	0.19

**Outcome**			
Death	6 (42.9%)	13 (38.2%)	0.77
Good outcome (mRS 0–3)	5 (35.7%)	14 (41.2%)	0.73
Duration of hospitalization days (median with range)	26 (1–79)	13.5 (4–73)	0.08
Duration of ICU stay in days (median. range)	17.5 (1–60)	4.5 (0–41)	0.002
MV	14 (100%)	17 (50%)	0.001
Duration of MV days (median range)	12 (1–60)	0.5 (0–21)	<0.001
VAP	8 (57.1%)	9 (26.5%)	0.17
Sepsis	11 (78.6%)	23 (67.6%)	0.55
Arrhythmia	1 (7.1%)	2 (5.9%)	0.46
Tracheostomy	6 (42.9%)	3 (8.8%)	0.01

*CNS, central nervous system; ICU, intensive care unit; mRS, modified Rankin Scale; MV, mechanical ventilation; STESS, status epilepticus severity score; VAP, ventilator associated pneumonia*.

## Discussion

In the present study, 13% patients with SE had SRSE and 43% of them died. The commonest etiology of SRSE was metabolic encephalopathy and CNS infections. None of the patients with SRSE had history of epilepsy. The patients in the present study have been included based on predefined inclusion criteria. In the Chinese study, there were two deaths in the hospital and four (36.6%) at 3 months. Old age and comorbidities predicted the poor outcome (6). In the French ICU survey on prolonged SRSE, out of 130 ICUs, 15 reported no patients, 19 had at least one patient fulfilling the inclusion criteria, and the remaining ICUs provided no information. The results were based on 78 patients (7). This study highlights the difficulty and limitations in obtaining information on SRSE. In the studies form South India, 16.9% and 7.8% patients had SRSE during 8 and 5 years, respectively. SRSE was significantly more common in the extremes of ages and was due to acute symptomatic etiology in 63.4% patients. The patients with encephalitis had a high chance of SRSE (8). The etiology of SRSE may have regional variation. In the study from China, 9 out of 13 patients were due to encephalitis; toxoplasma and cytomegalo virus in one each, and the etiology in the remaining patients was not confirmed. In the French study, there was diverse etiologies including epilepsy in 11 (14.1%), stroke in 10 (12.0%), metabolic encephalopathy in 5 (4.5%), encephalitis in 3 (3.0%) and neurodegenerative disorders, drug abuse, and post-surgery in 4 (5.1%) each. Categorizing SRSE on the basis of ILAE classification did not offer much advantage for general physician. Categorizing to potentially fatal or treatable disorders, however, may be advantageous.

Paucity of chronic epilepsy as a cause of SRSE has been highlighted in an earlier report (9). It is possible that the patients with epilepsy are under treatment and surveillance, which may account for their seizures to be treated better compared to those with new onset seizure. This was noted in our study as well as studies from China and South India (6, 8). The median duration of SE in our study was longer in encephalitis (240 h) compared to miscellaneous (168 h) and metabolic encephalopathy (48 h) groups. The patient with cerebral venous sinus thrombosis had SE for 432 h. The duration of MV in our study was longer in miscellaneous compared to metabolic and encephalitis groups. In the study from France, the median ICU stay after administration of general anesthesia was 17 days (7). In the study from South India, the mean duration of ICU admission and duration of MV was longest in children (8). In spite of such long duration of SE with higher frequency of MV, the mortality in our study was comparable with other reported series (43%) (Table 5).

**Table 5:** Comparison of different studies on super refractory status epilepticus.

Variables	Li et al. (9)	Lai et al. (10)	Jayalakshmi et al. (11)	Present study
Number of patients	13	78	30	14
Age in years	27 (16–81)	57 (36–70)	24.6 ± 23.6	27.5 (2–76)
Females	5 (33.3%)	35 (44.9%)	17 (56.7%)	7 (50%)

**Etiology**				
Epilepsy	2 (15.4%)	11 (14.1%)	0 (0%)	0 (0%)
Stroke	1 (7.7%)	10 (12.8%)	1 (3.3%)	1 (7.1%)
Encephalitis	9 (69.2%)	10 (12.8%)	20 (66.7%)	5 (35.7%)
Metabolic	0 (0%)	5 (6.4%)	2 (6.6%)	5 (35.7%)
Unknown	0 (0%)	27 (34.6%)	0 (0%)	0 (0%)
Others	1 (7.7%)	15 (19.2%)	3 (9.9%)	3 (21.4%)
Duration mechanical ventilation		–	22.2 ± 16.1	12 (1–60)
Burst suppression		48 (61%)		1 (7.1%)
Death	2 (15.4%)	41 (52.6%)	12 (40%)	6 (42.9%)

**Good outcome**				
Discharge	4 (30.7%)	9 (11.5%)	10 (33.3%)	5 (35.7%)
1 year	7 (53.8%)	12 (17.1%)		
Intensive care unit (ICU) stay (days)	16	28	29.7 ± 21.4	17.5
ICU complications			20 (66.6%)	
VAP	13 (100%)	27 (34.6%)		8 (57.1%)
Hypotension	1 (7.7%)	60 (76.9%)		13 (92.9%)
Sepsis	1 (7.7%)	60 (76.9%)		11 (78.6%)
Others	Metabolic acidosis (1) (7.7%)	ARF (14) (17.9%)		Tracheostomy 6 (42.9%)
				Arrhythmias 1 (7.1%)
Magnetic resonance imaging abnormal	6 (46.1%)	–		7 (50%)

In view of poor outcome in the patients with SRSE, ethical concerns have been raised on the cost effectiveness of ICU care in these patients (7). However, the recent reports have suggested that acceptable recovery is possible following aggressive treatment. In our study, the encephalitis patient especially those with a treatable etiology survived. On the other hand, SRSE patients with irreversible etiology such as dialysis-dependent uremic encephalopathy had poor outcome. It is important to identify the patients who will respond to aggressive therapy. The prognostic predictors in the retrospective studies included male gender, younger age, burst suppression within 7 days, treatable etiology, and lack of need of vasopressors. The ICU-related complications included hypotension, pneumonia, sepsis, and cardiac arrhythmia, and may contribute to the poor outcome. Hypotension was seen in 92.9% of our patients; however, it did not adversely affect the outcome.

Our study is limited by small sample size and high proportion of metabolic and infectious etiologies. We did not do continuous EEG monitoring in all the patients, which may have underestimated NCSE in our study. We have used burst suppression in one patient only. However, concern has been raised on the safety and efficacy of IV anesthetic drugs in SE. In a retrospective review of 171 patients, 37% were treated with IV anesthetics, which resulted in more infections (43 vs 11%) and 2.9-fold relative risk of death (20). In another study on 126 patients with SE, administration of IV anesthetic was associated with poor outcome (21). These studies raise the question whether IV anesthetic drug do more harm than good, and should we change our practice? (22). There is a need of a prospective study on SRSE.

## Ethics Statement

This study was approved by Institutional Ethics Committee, SGPGIMS, Lucknow, India.

## Author Contributions

Substantial contributions to the conception or design of the work; the acquisition, analysis, or interpretation of data for the work; and drafting the work or revising it critically for important intellectual content: UM, JK, DD. Final approval of the version to be published: UM.

## Conflict of Interest Statement

The authors declare that the research was conducted in the absence of any commercial or financial relationships that could be construed as a potential conflict of interest.
